# Intensive care staff, the donation request and relatives’ satisfaction with the decision: a focus group study

**DOI:** 10.1186/1471-2253-14-52

**Published:** 2014-07-11

**Authors:** Jack de Groot, Myrra Vernooij-Dassen, Anneke de Vries, Cornelia Hoedemaekers, Andries Hoitsma, Wim Smeets, Evert van Leeuwen

**Affiliations:** 1 Radboud Institute for Health Sciences, Radboud University Medical Center, Nijmegen, the Netherlands; 2 Department of Spiritual and Pastoral Care, Radboud University Medical Center, Nijmegen, the Netherlands; 3 Kalorama Foundation, Nijmegen, the Netherlands; 4 Department of Intensive Care Medicine, Radboud University Medical Center, Nijmegen, the Netherlands; 5 Department of Nephrology, Radboud University Medical Center, Nijmegen, the Netherlands; 6 Department of Religious Studies and Theology, Faculty of Humanities, Utrecht University, Utrecht, the Netherlands

**Keywords:** Organ donation, Qualitative research, Health care professionals, Donation request, Regret

## Abstract

**Background:**

Effectiveness of the donation request is generally measured by consent rates, rather than by relatives’ satisfaction with their decision. Our aim was to elicit Dutch ICU staffs’ views and experiences with the donation request, to investigate their awareness of (dis)satisfaction with donation decisions by relatives, specifically in the case of refusal, and to collect advice that may leave more relatives satisfied with their decision.

**Methods:**

Five focus groups with a total of 32 participants (IC physicians, IC nurses and transplant coordinators) from five university hospitals in the Netherlands. Transcripts were examined using standard qualitative methods.

**Results:**

Four themes (donation request perceived by ICU staff from the perspective of relatives; donation request perceived by ICU staff from their own perspective; aftercare; donation in society) divided into 14 categories were identified.

According to ICU staff, relatives mentioned their own values more frequently than values of the potential donor as important for the decision. ICU staff observed this imbalance, but reacted empathically to the relatives’ point of view. ICU staff rarely suggested reconsideration of refusal and did not ask relatives for arguments.

ICU staff did not always feel comfortable with a request in the delicate context of brain death. Sometimes the interests of patient, relatives and those on the waiting list were irreconcilable.

ICU staff were mostly unaware of relatives’ regret following their decisions. Aftercare did not provide this type of information.

Donation request by IC physicians was influenced by the way organ donation has been regulated in society (law, donor register, education, media).

**Conclusions:**

Our findings lead to the hypothesis that giving relatives more time and inviting them to reconsider their initial refusal will lead to a more stable decision and possibly more consent.

## Background

The effectiveness of the donation request is generally measured by consent rates [1-11]. Donation rates also seem to be the primary interest in research about attitudes of intensive care unit staff (ICU staff) [12]. However, donation rates are determined by relatives. The decision to donate or not may be extremely difficult. Publications addressing decision stability report substantial dissatisfaction, especially with donation refusal [13-16] and even traumatic memories regarding the donation request [4,17,18]. This research outcome does not seem to influence daily practice of ICU staff [19]. However, it does seem important, because preventing regret in cases of refusal can primarily lead to no or less harm for relatives and consecutively the possibility of more organs.

We carried out our research in the context of the Dutch law on organ donation. The aim of our research is to elicit ICU staffs’ views and experiences with the donation request, and to investigate their awareness of (dis)satisfaction with donation decisions by relatives, especially in cases of refusal. Our ultimate goal was to collect advice that could be used to produce a higher rate of satisfaction among the relatives.

## Methods

### Design and sampling

To elicit Dutch ICU staffs’ views and experiences with the donation request we conducted a qualitative study with a total of 32 ICU professionals, divided into five focus groups [20] from November 2010 until March 2011 (Table 1). Focus groups were chosen for data collection because, compared to questionnaires and one-to-one interviews, the interaction between research participants can encourage open conversation about sensitive subjects and facilitate the expression of ideas and experiences that might be left underdeveloped in an interview [21]. In cooperation with an ICU physician (CH) the primary investigator recruited 24 participants by general (email) invitation to all professional workers in the ICU of the University Medical Centre (UMC) Nijmegen, as well as 8 participants by a directed invitation of professionals from another 4 Dutch transplant centres (also UMCs) in the Netherlands. Participation was voluntary. Focus group meetings lasted between 67 and 94 minutes, with an average of 76 minutes. We organised two mono-disciplinary groups (nurses and medical specialists) and three mixed groups (nurses, physicians and transplant coordinators), expecting differences in openness.

**Table 1:** Characteristics of participants

	**Number/Years**	**%**
**Total**	32	100
**Gender**		
**• Male**	16	50
**• Female**	16	50
**Profession**		
**• Physicians**	12	37
**• Nurses**	15	47
**• Transplant coordinators**	5	16
**Age**		
**• 25-39 years**	11	34
**• 40-49 years**	13	41
**• 50+**	8	25
**Mean age total group**	43.4	100
**Experience in health care in years - mean**		
**• Physicians**	20.1	37
**• Nurses**	18.2	47
**• Transplant coordinators**	25.0	16
**Mean total group**	20.0	100
**Experience in ICU and/or organ donation in years - mean**		
**• Physicians**	12.0	37
**• Nurses**	10.0	47
**• Transplant coordinators**	8.0	16
**Mean total group**	10.5	100

Our study was approved by the UMC Nijmegen research ethics board.

### Focus group sessions

An experienced moderator (CF) facilitated the discussion and flow of the focus groups using a structured interview guide (Additional file 1), based on a review of current literature [22]. The interview guide was pilot tested with two physicians and two nurses to ensure that the guide was clear and well-understood.

Firstly, we initiated a discussion about participants’ attitudes to and experiences with the request for organ donation to relatives of a potential donor. In the second part we asked the participants to consider the decision making process and relatives’ possible regret about refusal, thereby focusing on possibilities for discovering and preventing this regret.

### Data collection and analysis

All focus group sessions were audio and video taped; one co-moderator (WGS/HL) was present to record field notes of reactions that were undetectable by recorders. All sessions were transcribed verbatim. We used a practical guide for applied research [23,24] for data collection as well as a mix of conventional and directed content analysis [25]. Data were coded from the transcripts using a process of open, axial and selective coding [24,26], using Atlas.ti©-software. Three investigators (JdG/WS/AdV) independently developed a coding scheme by identifying, labelling and classifying the primary patterns in the content. These study investigators discussed coding; disagreements that could not be resolved were adjudicated by the principal investigator. All codes have been presented in Table 2.

**Table 2:** Code book

**Themes**	**Categories**	**Codes and subcodes**
**Donation request seen by relatives**	Decision	1. Communication with ICU staff and among relatives about the decision and afterwards,
		2. Patient’s wish (not) known from earlier communication or donation register,
		3. Agreement between relatives about the decision,
		4. Organ donation by children
	Evaluation	5. Values of patient, respect for autonomy of patient,
		6. Values of relatives,
		7. Influence of religion on the decision,
		8. Regret about decision,
		9. How do relatives feel about the donation request,
		10. Objectives by relatives against donation
	Support	11. Enough time to decide,
		12. Enough understandable information to make a decision,
		13. What is good care for the patient and the relatives to make a decision,
		14. Which kind of support can relatives help to make the right decision,
		15. Which person (counsellor) can guide relatives to the right decision
**Donation request seen by ICU staff**	Requestor	16. Requestor: who is requestor, who assists in the request, relational aspects between relatives and requestor,
		17. Task of the requestor, information required about the donation procedure and about brain death to bring relatives to a decision,
		18. Skills required for the requestor, use of information (about patient’s wish or ideas of relatives) gathered by nurses, use of information from the donation register
		19. Attitude required for the requestor
	Context of the request	20. Where, when, timing, initiative of relatives before the request, acuteness of the situation,
		21. Decoupling
	Interest	22. Interest of relatives, interest of potential donor, interest of patients on waiting list – as theme in the donation request
	Brain death	23. Brain death (understanding by ICU staff, by relatives), difficulties in care for a brain dead patient, determination of brain death, apneu test
	Feeling comfortable with donation request	24. How do ICU staff feel about the donation request, if they are requestor, if they facilitate the request
	Personal ideas about organ donation	25. Personal ideas of ICU staff about organ donation, registration, donation law; change in ideas as a consequence of experiences with donation procedures
**After-care**	Care after death for relatives	26. Offering relatives contact and care after leaving the hospital, request by relatives for aftercare
	Themes in aftercare contact	27. Themes of aftercare conversation, review of the donation decision including regret
**Organ donation in society**	Law and organ donation	28. Donation law, Dutch system (opt-in) in contrast to Spain, Belgium (opt-out)
	donation register	29. Donation register: how it works, preferences, motivation for registration, campaigns for registration, the importance of registration
	Organ donation as societal theme	30. Organ donation in media, education, information campaigns, in societal groups (family, health care, school)

All co-authors discussed the analysis and the findings and contributed to the discussion.

## Results and discussion

### Differences between focus groups

When ICU doctors spoke separately they paid more attention to their own perspective, discussing their requestor tasks and skills and the effects of their relationship with relatives. When nurses discussed organ donation separately, they revealed more of their own personal opinion as well as more criticism of the interventions of ICU doctors. They felt that some doctors requested for donation too early, e.g. before mentioning a possible brain death, and took too little time to stay with relatives once the question was raised and/or the decision was taken. In mixed groups (physicians, nurses, transplant coordinators) organ donation in society and law and the interests of different groups (potential donors, relatives, potential recipients) were more frequent topics. In the focus discussion transplant coordinators also commented on situations in which they had not been involved, such as when relatives initially refused donation and for that reason had no contact with the transplant coordinator.

### Interview themes

We identified four themes, divided into 14 categories, resulting in 30 codes (Table 2).

The first theme concerned the ICU staff taking the perspective of the relatives, the second theme concerned the experience of ICU staff from their own perspective, i.e. the perspective of the requestor, including their personal attitude towards organ donation and transplantation. The third theme concerned participants discussing the care after discharge. The fourth theme dealt with how ICU staff considered donation as a societal theme (legislation, media, education). We have presented some illustrative quotes (marked with Qn) for the categories in Table 3.

**Table 3:** Overarching themes

**Theme**	**Speaker**	**Qn**	**Quotation**
	**Profession, gender, age**		
**Donation request seen by relatives**	MD m 52	1	The decision *‘Yes, I’ll do it’* is generally more carefully considered [than a refusal (addition JG)]. Also now, when we have to decide in advance, you take more time to think about a ‘yes-decision.
	RN m 43	2	I also notice that actually the question *‘Why not?’* is never really discussed in more detail. What are really the weighty arguments not to do it? And nine, even ten out of ten times the conversation is finished then.
	TC f 49	3	No, the only regret that you hear occasionally is: *’If I had known this, that it would take so much time, that so many things needed to be arranged, I probably would not have done it’.*
	MD m 57	4	That is what you frequently hear from parents: *‘Our child could not be saved, but he can save other children or other adults.’* It provides a kind of comfort for those people. There is an aspect of comfort included’.
	TC f 47	5	Whereas, and this is how I always explain it to our doctors and then I always say…, you should not give a point in time at all. Just say: *‘Take your time and think about it’.*
	TC f 47	6	That is a bit of a slogan of mine, which also, indeed, means the right information at the right moment, which also means well-informed and well-balanced. Sometimes you have to repeat it all, because the information has not yet sunk in, which is of course understandable; these people are in an acute and stressful situation and are perhaps not able to fully take in all this information.
	MD m 52	7	I think the most difficult thing is to find a balance, especially in an acute situation, between the care and the relatives’ grief and your own feeling of the best care you want to give this family. You need to gather all your emotions to make such a sudden death bearable for the relatives. At the same time and completely contrasting is the importance of the organ, needing to be preserved. That is for me the most difficult thing, because what you would rather do is concentrate on one thing, namely guiding the relatives in such a terrible period. That is difficult.
	TC f 47	8	Something that I also find very important, which is also a bit like stating the obvious, that there is someone present who is capable of giving optimal guidance, also with respect to time.
**Donation request seen by ICU staff**	MD m 52	9	It makes a great difference whether they have had the opportunity to build up a bond of trust with the relatives, because that makes the conversation a much easier one. I immediately admit that if you are confronted with a family for whom this has suddenly happened, and you do not know them, I think it is still one of the most difficult conversations to have.
	MD m 34	10	What happens sometimes is that you go for the heart-beating procedure, but it takes too much time to complete the brain-death-protocol, which leads you to say: there is a second option, let us stop this now. In other words you stop the treatment, not for the patient himself, but to go for the non-heart-beating procedure.
	MD m 58	11	I think it would be a good thing to train people’s communication skills. That would do a great deal of good. I have had to make my own way in this, and I do feel that I have succeeded, but I also think that this is difficult for younger colleagues.
	TC m 50	12	These are funny things you hear afterwards: *‘We already knew how bad the situation was and we have already been thinking about it’*. Especially with parents it is incredible to see how they are able to empathise with parents of other seriously ill children, who are waiting for a transplantation. These parents feel that if their child could save another child, that would be wonderful. To be able to set aside their own grief and go to the doctor or nurse with that in mind and say: *’Well, if we can be of any help by giving consent, we will do that’.* And no one has even brought up that question yet.
	TC f 49	13	You should not victimise the people who are left behind. We tend to exaggerate sometimes, the donation request is terribly difficult and if you say ‘yes’ or ‘no’, it is so hard….. Mourning is normal, losing someone and mourning their loss is normal, I think. You can cause damage with a donation request, if you do it in a tactless way or the request is rather wrongly timed, in so far as you can time such a thing, but that is what I think.
	MD f 42	14	If the family says ‘no’, while the patient has consented, that is an extraordinarily inconvenient situation, especially because the family is very vulnerable and for that reason I would not dare to put more pressure on them.
	TC m 50	15	I am a transplant coordinator, although I feel that I am a donation coordinator. I am there for the relatives, for the donor, and for the ICU staff too. I am there to assist all of these people in their weighty task by guiding them through this procedure. That is my intention, yes. Obviously I sympathise very much with all of the people who are on the waiting list for organ transplantation, and I hope that they will receive an organ of good quality.
	TC f 47	16	I always say…try to find out why they cannot accept the patient’s will. Sometimes it can be just a tiny thing, often fear, which is not to say that that is a small thing but it can be something that can be solved… through proper counselling. Or sometimes it is something else…, *‘not the heart, but the rest is alright’*. So it is as if you are striking a bargain, as you just said. Yes that sounds very familiar.
	MD m 33	17	You tend to give priority to the emotions of the relatives rather than to the will of the patient…..
	MD m 57		You are afraid of having a difficult conversation, you are the doctor, right? Because then you feel you have to act like some sort of body snatcher, trying to coax the organs out of the body.
	RN f 32	18	I had been registered from the age of eighteen. But since I learned about the length of the procedure, I would rather let my family decide about it, because I think it will create a heavy burden for them.
**Aftercare**	RN f 41	19	If you ask them whether they feel regret, what would people have wanted otherwise, well, that bit you do not know about.
	MD m 50	20	You often know whether a request was made, but that is it. If it is ‘yes’, they always get a message from the transplant coordinator and often a meeting, and if it is ‘no’ then it is no and we do not follow up on it.
	TC f 47	21	But if you said ‘no’, and you have a meeting afterwards with your doctor, especially if you regret your decision, that makes it even harder to talk about the subject.
**Organ donation in society**	MD f 37	22	Now at least we know that this is not actually working very well. Our system….
	MD m 52		In Belgium the system is such that you really need to think about it. Because you have to come to a decision and that decision will be carried out.
	MD m 58	23	I am there for the patient. So my point is….. the patient has in fact signed a testament, that’s how I communicate it to the relatives.
	RN f 34	24	I once had a conversation with relatives who said*…, I just feel that they are kind of shopping with organs, with all that media coverage……*. I feel that it is difficult to try and prevent that.

### Theme 1: Donation request perceived by ICU staff from the perspective of relatives

ICU staff not only tried to imagine the impact of the donation request on the relatives, but also how relatives decide, how they evaluate their decision and what is helpful in making that decision. In general, ICU staff knew more about the result than about the process of decision making. They respected refusal by relatives, but were not usually aware of how (dis)satisfied relatives were with that decision, especially after refusal. In the following paragraphs we will present different categories mentioned by ICU staff taking the perspective of the relatives in more detail.

#### Decision

Decision making was described as particularly difficult for relatives when the patient’s wish was unknown and/or if relatives disagreed with each other about the decision. Refusal seems to be considered in a less serious way by relatives than consent (Q1). ICU staff said that they seldom encouraged relatives to reconsider their decision. ICU staff also explained that relatives sometimes gave consent before the request was made.

#### Evaluation

ICU staff observed that patients’ values and religion were scarcely mentioned as an argument in the decision, yet objections and values of relatives played a much more important role. ICU staff rarely asked for arguments justifying refusal (Q2). So ICU staff cannot foresee regret, except from those who consented and were surprised by the length of the procedure (Q3). ICU staff said they could understand these objections of relatives and that this could be a reason for refusal.

Values of relatives were seen as important, especially for parents, because they give meaning to the perceived meaningless death of their child (Q4). Also for other relatives some comfort was attributed to donation.

#### Support

To arrive at a good decision it would seem to be necessary that relatives have enough time (Q5), are adequately informed (Q6) and receive the best possible care (Q7). A plea was made for an exempt professional to guide the process (Q8). The ICU staff had never before considered a more specialised form of guidance in decision making, nor support by an independent counsellor, thus rejecting the suggestion to introduce such a professional.

### Theme 2: Donation request from the perspective of health care professionals

ICU staff said that they did not always feel comfortable with a request. The requestor has to deal with, in their eyes, the seemingly irreconcilable interests of the patient, the relatives and those on the waiting list. The request is a collaborative process between doctor and nurse, with the possibility of inviting the transplant coordinator for a detailed explanation. In the following paragraphs we will elaborate more on the different categories from the perspective of the ICU staff.

#### Requestor

The donation request is made by the physician treating the potential donor. He is assisted by nurses, who, –as participants said–, play an important role in the total requesting process. Sometimes they are able to inform the physician, prior to the request, about the views of the patient or the relatives on the subject of donation*.* The relation between requestor and relatives is seen as important; ‘trust’ is a key word (Q9). It is important to identify a patient as a potential donor. Although physicians said that they usually tend to go for the maximum outcome (donation after brain death), they said that they would also be content with an optimum outcome: if donation after brain death is impossible, one can try to go for a donation after circulatory death (Q10). The most important task is to provide clear information on the patient’s situation and specifically to explain brain death. The donation request requires special communication skills and attitude, especially empathy. Training and experience were highly recommended to deal with the request task (Q11).

#### Context

ICU staff usually underscored the principle of decoupling. They deliberated on when a donation request could best be made: after a complete brain death procedure or based on the clinical view. If relatives brought up the issue of donation themselves, the request could be made at an earlier stage as opposed to in a situation where relatives express apathy as a mourning reaction (Q12).

The context in which the question was raised was described as extremely delicate. As one physician put it: it is always *‘the most difficult question at the most difficult moment; besides it is very emotional for both the requestor and the relatives’.* At times, this made it inconvenient in a sense for the requestor. ICU staff were aware that the donation request could give rise to a lot of emotions. The request was often made to people who were considered unable to think clearly because they were exhausted, although this should not be a reason for not raising the question. The decision of the relatives depended on the timing of the request (Q13).

#### Interests

ICU staff have to deal with the interests of the patient, the relatives and also those on the waiting list. Sometimes these interests seem irreconcilable, especially when relatives refuse donation in spite of a donor registration. In most cases, doctors and nurses accept the decision of the relatives (Q14). Transplant coordinators explicitly stated that they considered all interests (Q15). Transplant coordinators recommended a person-oriented approach: try to discover relatives’ motives for refusal, find out if they are capable of making a decision, consider their need for empathy, counselling, information and quiet or rest, and continue caring for their beloved one with respect and dignity. Rather than treating relatives as victims (Q13), transplant coordinators were more inclined to negotiate with them (Q16).

#### Brain death

Brain death is a difficult concept to understand and to explain, even for ICU staff. It would seem better to make the donation request after the diagnosis of brain death, than after the prognosis of brain death; after such a prognosis relatives sometimes chose donation after circulatory death, instead of waiting for donation after brain death to become possible (Q10).

#### Feeling comfortable with the donation request

ICU staff said they felt uncomfortable with relatives refusing donation in cases of positive registration. A tension existed between acting on legal, ethical or humanitarian grounds. ICU staff expressed their solidarity with the patient and insisted on the autonomous choice of the patient in cases of positive registration. They often reacted empathically to the viewpoint of the refusing relatives (Q17).

Some members of the ICU staff said they experienced the donation request as extremely difficult. Others said that it might be more difficult for the ICU staff than for the relatives*.*

#### Personal opinions regarding organ donation

ICU staff remarked that the practice of organ donation can lead to an affirmation of one’s personal ideas about donation, yet it can also lead to a change of opinion, especially for nurses. In fact, some withdrew their registration as a donor (Q18).

### Theme 3: Care for relatives after discharging

Relatives who gave their consent received more aftercare (on behalf of the transplant coordinators) than those who refused. ICU staff therefore thought that they ran a lesser risk of being informed about regret in cases of refusal (Q19). Even if ICU staff did have contact they did not ask about possible dissatisfaction with the refusal by the relatives (Q20). Also, relatives did not mention regret spontaneously because that seemed to be a difficult thing to do (Q21). So the subject remained undiscussed.

### Theme 4: Organ donation in society

The donation request is influenced by the way organ donation has been regulated in society (law, regulation by the donor register, education, media).

#### Law and organ donation

ICU staff compared the Dutch legal systems (opt-in) and noticed in discussion advantages in the Belgian system (opt out) (Q22).

#### Donation register

ICU staff pleaded for an obligatory system of registration (Active Donor Registration), which was conceived as a testament (last will) of the patient. They will carry out this last will (Q23).

#### Organ donation as a societal theme

ICU staff observed a lot of prejudices and a lack of information about organ donation in society (Q24).

### Discussion

Our study partly confirms findings of earlier research [22]. ICU staff were informed about nearly all factors that influenced the donation decision in the direction of consent: timing the request, decoupling, experienced requestor, empathic attitude, explanation of brain death, knowing the patient’s wishes and (dis)agreement amongst relatives. They knew which measures to take to support relatives in their decision making: giving time and providing information to come to a decision, giving good care and attention to patient and relatives [27]. Even so, transplant coordinators observed that some physicians gave relatives the impression that they had to decide rapidly. So in real life the best practice was not always carried out.

A new aspect arising from our research is that Dutch ICU staff seldom encountered regret from relatives about their donation decision. ICU staff were not familiar with the aforementioned decision stability literature [13-16]. Moreover, from their own practice they did not know whether relatives were (dis)satisfied with their decision. Even when staff did have aftercare contact, the motto seemed to be: “Don’t ask, don’t tell”. ICU staff only assumed regret in cases where the patient’s wishes had been expressed in the donor register and relatives had gone against these wishes. Most physicians were inclined to retire from the consultation room following a refusal, hoping to prevent traumatic memories by not insisting too much on consent. Transplant coordinators were more inclined to ask for motives for refusal and to negotiate about the objections, but are seldom called in after initial refusal.

Remarkably, ICU staff mainly focused on cases in which relatives refused donation, despite their beloved one having been registered as a donor. ICU staff did not seem to be aware that this happens only in exceptional cases :6% at the average in the years 2009-2012, that means approximately five cases each year in the Netherlands. Less exceptional (67%) are refusals when the donor was not registered or the register was not consulted (14%). As far as we know no data have been published in the Netherlands about regret after refusal. Unpublished data from an earlier mentioned research project [27] revealed 19% doubt or regret following refusal (11 out 57). If we use this percentage as indicative, one can assume that focusing on the group who tend to refuse in an uncertain situation ‘might deliver more organs’ in the Netherlands, perhaps more than 50 donors each year. However, our interest is not primarily to increase consent rates, but to make more relatives feel satisfied with their decision. We assume that paying more attention to the decision process can serve both intentions: less regret, more organs.

Interestingly, only wishes expressed in the donor register were mentioned as a source of information. Yet donation intentions can also be determined from private conversations or be attributed to the potential donor, derived from his lifestyle and value system [28]. This strategy of discovering patients’ donation preferences had not been discussed in the focus groups. As mentioned, relatives were seldom asked why they had refused donation and were not encouraged to reconsider their decision. However, if relatives were encouraged to (re)consider the values of the patient, it might be possible to discuss how organ donation can fulfil those values. This approach, where more attention is given to the values of the potential donor as attributed by the relatives, has not yet received full attention, and might, as we hypothesise, lead to a more stable decision and maybe even more cases of consent.

#### Advice

Although our focus group participants were unaware of the number of relatives who may regret their refusal, they made at least four remarks, which may serve as advice. First, they underscored the suggestion in a recent thesis [29], not to request a donation too quickly. Although decoupling as a concept is more than 20 years old [14], it is not general practice in the Netherlands [30]. In fact, brain death determination often does not happen at all, because when a request is made too soon, relatives refuse donation in advance [31,32]. Second, transplant coordinators in our focus groups advised concentrating on communication with relatives, rather than insisting too much on donation. These suggestions have been affirmed by research, which demonstrated that support from a trained donation professional, who was not part of the treatment team, led to more consent in a natural way. Time spent with the family in combination with such a well-trained donation professional made the family decision to consent or refuse donation a well-considered one (as well as proving to have a positive impact on the family consent rate) [27,29]. Although collaborative requesting [1,2,11] is not a practice in the Netherlands, it might well be considered. Third, giving time, as suggested by some participants in our research, is important and is underscored by literature mentioning that initial decisions are rarely withdrawn [7], many refusals are not based on deeply held views [14] or correlate with the feeling of not having enough time to discuss donation [16].Finally, as one participant noted, *‘being comfortable with the request’* means not giving the impression of being stressed and not pressing the relatives into making a rapid decision.

So giving relatives time and room to decide without pressure, yet asking them to (re)consider the values of the patient and how organ donation can fulfil those values might, as we hypothesise, contribute to less relatives regretting and more organs. This hypothesis deserves further investigation.

#### Strength and constraints

The approach with monodisciplinary focus groups and mixed groups gave us differentiated information on the three professional groups engaged in the donation request. Participants work in UMCs covering around 80% of all Dutch transplantations. As this is a study of ICU staff in five transplant centres in the Netherlands, one cannot generalise the findings in other situations in which the donation request has been made. Some advice has been directed at the Dutch situation, whereby the donation request was usually done by the treating physician prior to official brain death clarification [32]. Other proposals in this study might also have advantages in other countries.

## Conclusions

ICU staff were not aware of (dis)satisfaction with donation decisions by relatives. On the whole they accepted relatives’ decisions, even if those relatives did not follow the patient’s wishes. ICU staff were aware of nearly all factors influencing the donation decision in the direction of consent, and they knew which measures were necessary to support relatives in their decision. We hypothesise that giving more time and inviting relatives to reconsider their initial refusal will lead to a more stable decision and possibly more consent.

## Abbreviations

ICU: Intensive care unit; PICU: Paediatric intensive care unit; UMC: University Medical Centre.

## Competing interests

The authors declare that they have no competing interests.

## Authors’ contributions

JG, MVD, WS and EL coordinated the design of the study and managed the project, JG, MVD, CH and WS organised the focus interviews, JG, MVD, AV and WS did the coding process and data analysis, JG and MVD wrote the draft of the manuscript, all authors contributed to the discussion and read and approved the final manuscript.

## Authors’ information

JG, WS and AV are hospital chaplains, CPE supervisor of the Department of Pastoral and Spiritual Care. They all have a degree in theology and are ordained as ministers in their own church.

Moreover, JG has a degree in bioethics and social sciences, WS (PhD) has a degree in social sciences, and AV (PhD) has a degree in linguistic sciences.

MVD and EL are Professor (PhD) in medical sociology (MVD) and medical ethics (EL), specialists in qualitative research at Nijmegen Centre for Evidence Based Practice, Radboud University Medical Center.

CH (MD, PhD) is head of the neurological ICU of Radboud University Medical Center.

AH (MD, PhD) was scientific director of the Dutch Transplant Foundation and Professor at Radboud University Medical Center.

In their daily work, JG and CH are engaged with eligible brain dead donors and their relatives.

## Pre-publication history

The pre-publication history for this paper can be accessed here:

http://www.biomedcentral.com/1471-2253/14/52/prepub

## Supplementary Material

Additional file 1 Interview guide. Click here for file
