# Supplementary material for: Intensive care staff, the donation request and relatives’ satisfaction with the decision: a focus group study
Source: BMC Anesthesiol. 2014 Jul 11;14:52. doi: 10.1186/1471-2253-14-52 (PMC4107587; doi:10.1186/1471-2253-14-52)
Supplement: Additional file 1 — Interview guide. [file 1471-2253-14-52-S1.doc]

Additional file 1

| Interview guide |
| --- |
| - **Associations** Which associations surface at the term “organ donation”? *(technical, organisational, emotional etc.)* |
| - **Circumstances** What are the experiences with the circumstances around the donation request?  *(after a period in which the doctor has tried to save the patient, as a result of which the relatives are hopeful, the announcement is made of the (impending) death of the patient ((brain death; shift from patient care to donor care; circumstances which make it difficult or impossible to request for donation)): how do ICU staff feel about that?)* |
| - **Experiences** Have doctors/nurses encountered difficulties, feelings of hopelessness or emotions by the relatives in answering the donation request? *(Do they recognise the relatives having difficulties with the request, do they observe disagreement between relatives, moral distress, existential questions; are moral and existential questions expressed?)* Are doctors/nurses comfortable with the decision making and the ultimate decision of the relatives? *(reasons, reactions to those feelings, suggestions for improvement)* |
| - **Regret** Do doctors/nurses meet relatives who afterwards regretted their decision? *(do they follow up on the relatives’ decision? When? Reactions?)* What type of contribution can help in relatives not regretting their decision afterwards? |
| - **Offer of counselling** Has the possibility ever been considered to offer a kind of coaching / counselling for decision making? Significance of that kind of support – who has to provide this kind of support? *(Suggestions for improvement of quality of care for relatives who experience difficulties in decision making).* |
